# Focusing analytes from 50 μL into 500 pL: On-chip focusing from large sample volumes using isotachophoresis

**DOI:** 10.1038/s41598-017-10579-5

**Published:** 2017-09-05

**Authors:** Xander F. van Kooten, Marianna Truman-Rosentsvit, Govind V. Kaigala, Moran Bercovici

**Affiliations:** 1 Faculty of Mechanical Engineering, Technion – Israel Institute of Technology, Haifa, 3200003 Israel; 2 IBM Research – Zurich, Säumerstrasse 4, 8803 Rüschlikon, Switzerland

## Abstract

The use of on-chip isotachophoresis assays for diagnostic applications is often limited by the small volumes of standard microfluidic channels. Overcoming this limitation is particularly important for detection of ‘discrete’ biological targets (such as bacteria) at low concentrations, where the volume of processed liquid in a standard microchannel might not contain any targets. We present a novel microfluidic chip that enables ITP focusing of target analytes from initial sample volumes of 50 μL into a concentrated zone with a volume of 500 pL, corresponding to a 100,000-fold increase in mean concentration, and a 300,000-fold increase in peak concentration. We present design considerations for limiting sample dispersion in such large-volume focusing (LVF) chips and discuss the trade-off between assay time and Joule heating, which ultimately governs the scalability of LVF designs. Finally, we demonstrate a 100-fold improvement of ITP focusing performance in the LVF chip as compared to conventional microchannels, and apply this enhancement to achieve highly sensitive detection of both molecular targets (DNA, down to 10 fM) and whole bacteria (down to 100 cfu/mL).

## Introduction

Isotachophoresis (ITP) is an electrophoretic technique that simultaneously focuses and separates ions based on differences in their electrophoretic mobility^1^. Although on-chip implementation of ITP has found use in a variety of applications, including protein focusing^2^, nucleic acid purification^3^ and bacteria detection^4^, its applicability to low-concentration targets is inherently limited by the small internal volume of microfluidic channels (typically on the order of 100 nL). This limitation is even more critical for large, discrete targets, such as bacteria, as their presence in the sample volume processed by ITP may become probabilistic, so that no bacteria are focused although they are present in the sample. To enable sensitive assays, it is therefore of critical importance to increase the volume of sample from which analytes are focused.

Preparative applications of ITP primarily involve separating and extracting targets from complex biological samples, such as whole blood^3^ and urine^5^. Preparative ITP has traditionally been performed in capillary electrophoresis (CE) machines capable of processing sample volumes on the order of ~10–100 μL^6–8^ using long capillaries (~1 m) and high electric fields (10–100 kV/m). In such systems, the use of larger-diameter capillaries contributes to large processed volumes, but must be balanced against the difficulty of removing the excess Joule heat generated in such channels. Recently, Marshall *et al*. translated ITP-based sample purification to an on-chip format, and demonstrated extraction of nucleic acids from 25 μL samples^9^.

Analytical applications of ITP primarily use ITP’s focusing capabilities to enable detection of low-concentration analytes. In plateau-mode ITP the initial concentration of analytes is sufficiently high for contiguous ‘plateau’ zones of focused and separated analytes to form between the leading (LE) and terminating electrolyte (TE). The sensitivity of such assays cannot be improved solely by increasing the cross-sectional area of the channels, as the focused sample is then also spread over an equally larger area^10^. This led Everaerts *et al*.^6^ to apply column coupling to plateau mode ITP, serially connecting a large-diameter capillary to a small-diameter capillary in order to achieve elongation of the ITP zones and improved resolution. This principle was later applied to large-volume (1 mL) processing in capillaries^11^ and translated to an on-chip format by Graβ *et al*.^12^.

In peak mode ITP, which is commonly used in biomarker detection, the sample concentration is too low to create a plateau zone, and the sample instead focuses at the narrow interface between the LE and TE. Here, too, the principle of varying cross-sectional area is effective, but instead of elongation of the zones, the concentration of the focused sample increases^2^. However, to date, demonstrations of column coupling in peak-mode ITP remain limited to devices with small internal volumes (<1 μL), presumably due to limitations in Joule heating and sample dispersion, which arise from scaling. As a result, while million-fold focusing was demonstrated by Jung *et al*.^13^ for a dye in unbuffered conditions, the highest reported focusing ratio for biological samples on-chip is only 10,000-fold, and the scaling constraints for designing chips capable of focusing larger volumes have thus far not been developed or studied.

To use the full potential of ITP as an analytical method, we present here the design of a planar microfluidic chip that enables 300,000-fold peak focusing of analytes contained in an initial sample volume of 50 μL under conditions compatible with biological samples, and demonstrate its benefits to ITP-based assays.

## Design considerations for large-volume focusing devices

**Figure 1:**
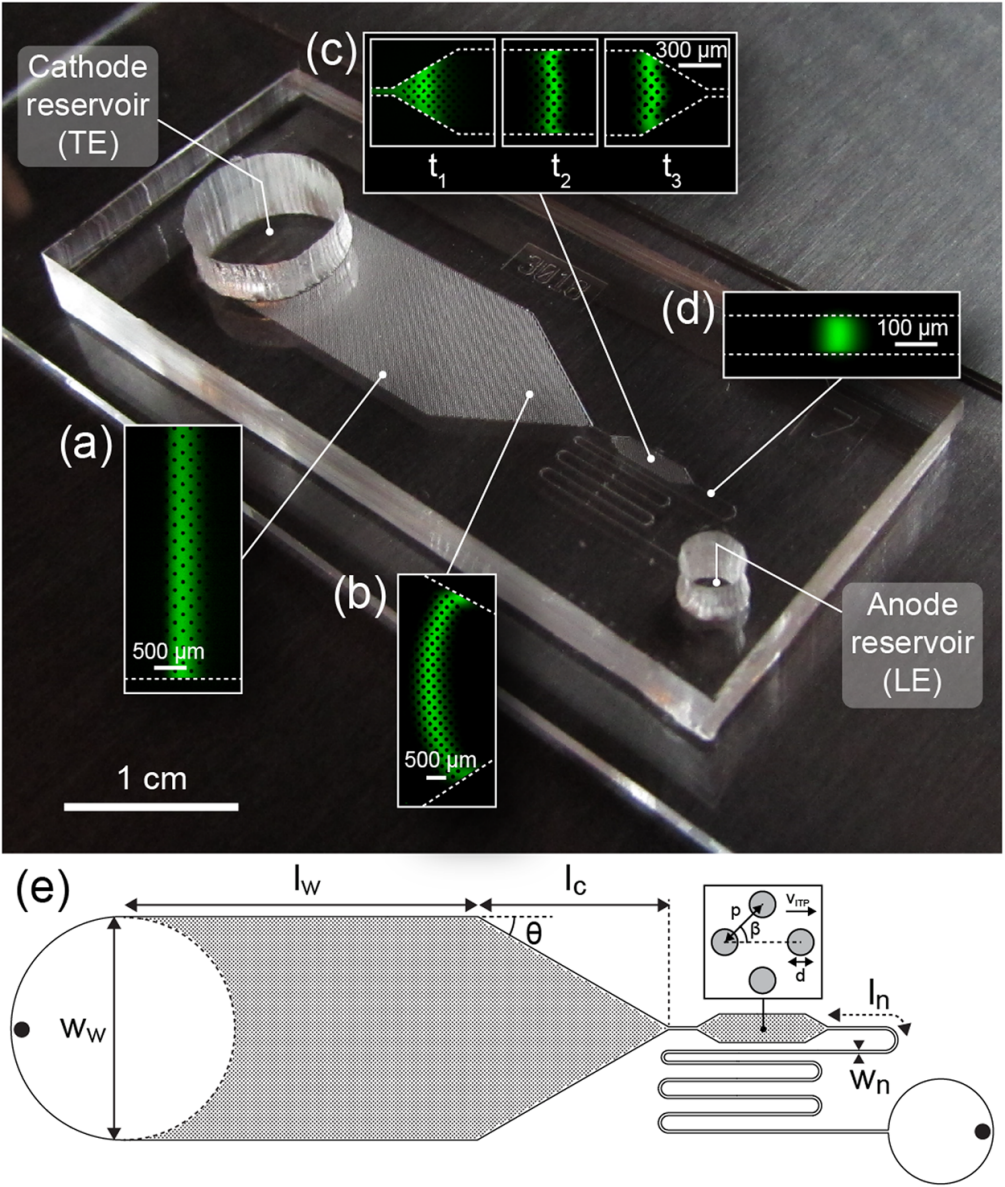
Photograph of a large-volume ITP device. Insets show a focused fluorescent dye. (**a**) The analyte first focuses uniformly across the entire width of an 8 mm wide region. (**b**) The wide region tapers down to a width of 100 μm, providing additional geometrical focusing. (**c**) An intermediate chamber geometry enables smooth refocusing of the interface from its dispersed state. (**d**) In the final narrow region, the ITP interface rapidly achieves its steady focused state before detection. (**e**) Top-view schematic of the device. Our primary device has a depth of 30 μm and dimensions *w*_*w*_ = 8 mm, *l*_*w*_ = 1.2 cm, *w*_*n*_ = 100 μm, *l*_*n*_ = 2 cm, *θ* = 30°, *l*_*c*_ = 6.5 mm, and uniform array dimensions *d* = 50 μm, *p* = 100 μm and *β* = 45°. Accounting for the occlusion of the channel by pillars, the effective width and length of the wide region are *w*_*w,eff*_ = 5.25 mm and *l*_*w,eff*_ = 8.2 mm, respectively. The location of electrodes is indicated by the filled circles in the reservoir (see Supplementary Information section S3).

Figure 1 presents the geometry of our large-volume focusing chip. The entire chip has a uniform depth of 30 μm to support a single-mask microfabrication process, and consists of four key regions: a wide channel containing an array of pillars where a large sample volume is processed (Fig. 1a), a narrow channel where the final detection takes place (Fig. 1d), a converging section connecting the two (Fig. 1b), and a chamber that enables the refocusing of the dispersed ITP interface to a sharply focused state after the convergence (Fig. 1c). The progression of the ITP interface through these regions is shown in the Supplementary Video S1.

A key point in the design of LVF chips is the control of dispersion throughout the path of the ITP interface. If the sample arriving at the entrance of the narrow channel is dispersed, the high electric field in the narrow region causes fast electromigration of the front part of the focused sample while the rear lags behind. This results in further dispersion of the ITP interface to the extent that the front part may reach the leading electrolyte reservoir before the rear fully enters the channel, and the analyte is unable to re-focus as it transitions through the narrow channel.

We identified two main sources of sample dispersion. First, electromigration in the wide region of the channel is susceptible to electrokinetic instability, leading to observable spatial frequencies developing in the lateral direction (see Supplementary Fig. S1). Zhang *et al*.^14^ used an array of posts to increase the hydrodynamic resistance of a wide channel in free-flow electrophoresis. Similarly, Liu *et al*.^15^ showed that the use of porous structures in a capillary significantly reduces dispersion caused by counterflow in ITP. As shown in Fig. 1a, we use a staggered array of cylindrical pillars with a diameter of 50 μm and a center-to-center pitch of 100 μm to reduce the characteristic lateral dimension for viscous action, resulting in a straight ITP interface throughout the section (see Supplementary Figs. S1 & S2).

The second cause for dispersion is the radially decaying electric field near the entrance to the narrow channel section, which leads to a curved ITP interface and ‘legs’ leading along the sidewalls (see Fig. 1b). This effect is worsened by Joule heating in the narrow section. The intermediate chamber shown in Fig. 1c allows the sample to refocus and enter the final narrow channel uniformly (see additional designs tested in Supplementary Figs. S3, S5 and Supplementary Table S1), after which the length of the ITP interface is once again determined by the balance between diffusion and electromigration.

## Results and Discussion

### Joule heating and assay time in large-volume ITP devices

As illustrated in Fig. 1e, consider a planar LVF channel of depth *H*_*c*_ comprising a wide-channel section of effective width *w*_*w,eff*_ and effective length *l*_*w,eff*_ filled with an electrolyte with conductivity *σ*_*w*_, connected in series to a narrow-channel section of width *w*_*n*_ and length *l*_*n*_ containing an electrolyte with conductivity *σ*_*n*_. The ratio of the electrical resistances of the wide and narrow channel is then ${R}_{w}/{R}_{n}={\sigma }_{n}{w}_{n}{l}_{w,eff}/{\sigma }_{w}{w}_{w,eff}{l}_{n}$. The relative resistance of the wide section reaches a maximum when the ITP interface is at the border between the two sections. As the conductivity of the adjusted TE is typically around 10% of that of the LE (for the chemistry used in this work, ${\sigma }_{LE}/{\sigma }_{TE}\approx 15$), the narrow channel continues to account for more than 85% of the voltage drop $V$ at all times (see Supplementary Information section S4). Hence, during electromigration of the ITP interface through the wide section of the channel, the electric current can be assumed constant, $I=V{w}_{n}{H}_{c}{\sigma }_{LE}/{l}_{n}$.

As the velocity of the interface in the wide region is much lower than in the narrow region, the migration time through the wide region will dominate the total time required to perform an assay. In the following, we therefore consider only the wide region. The current in the system can be assumed constant, so that the electric field in the LE is ${E}_{LE}=I/({\sigma }_{LE}{w}_{w}{H}_{c})=V{w}_{n}/({w}_{w}{l}_{n})$ and the time for the interface to reach a location $x$ in the channel is simply $t(x)=x/{\mu }_{L}{E}_{LE}=(x{l}_{n}/{\mu }_{L}V)({w}_{w}/{w}_{n})$, where μ_L_ is the effective electrophoretic mobility of the LE. Substituting $x={l}_{w}$, the time required for the interface to migrate through the wide channel becomes

1 $${t}_{A}=\frac{{l}_{w}{l}_{n}}{{\mu }_{L}V}\frac{{w}_{w}}{{w}_{n}}$$

At the same time, heat generation per unit volume is governed by the narrow channel, where the electric field is highest. On the timescale of electromigration, the device reaches thermal equilibrium, and following a similar analysis to that presented by Zehavi *et al*.^16^, the temperature increase is governed by a balance between Joule heat generation and dissipation through the glass bottom of the chips to the microscope stage, which is assumed fixed at room temperature (see additional information in Supplementary Information Section S5)

2 $${\rm{\Delta }}T=\frac{{\sigma }_{LE}{H}_{c}{d}_{glass}}{{k}_{glass}{l}_{n}^{2}}{V}^{2}$$

**Figure 2:**
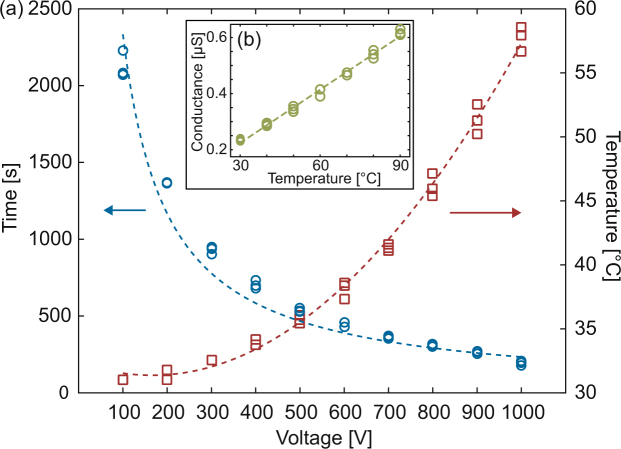
Total migration time and maximum temperature in the narrow region of an LVF device. (**a**) The operating voltage of a large-volume device determines the trade-off between assay time (left axis) and maximum temperature (right axis). (**b**) Calibration measurements of channel conductance at forced temperatures, which we used to map conductance values to temperature.

Figure 2 presents experimental measurements of the assay time and the maximum temperature in the narrow region of our LVF chip as a function of the applied voltage. As expected, the assay time is inversely proportional to the applied voltage, whereas the maximum temperature in the narrow region scales quadratically with voltage. The dashed lines show the best fit to 1/V and V^2^ dependences, with R^2^ values of 0.961 and 0.998, respectively. The fit of the temperature as a function of voltage yields a second-order polynomial coefficient (for the V^2^ term) of p_1_ = 3.73 × 10^−5^. Substituting *σ*_*LE*_ = 1.01 S/m, *H*_*c*_ = 30 μm, *d*_*glass*_ = 1 mm, *k*_*glass*_ = 1 Wm^−1^K^−1^ and *l*_*n*_ = 2 cm into equation (2) yields p_1_ = 7.1 × 10^−5^, indicating that the model provides a good quantitative estimate of the scaling, and enables order-of-magnitude predictions of the temperature in the narrow channel, though clearly 2D effects of heat dissipation into the PDMS result in fast heat removal in practice.

For some applications, such as immunoassays, it is important to maintain a sufficiently low temperature (typically <40 °C) in the channel to avoid denaturation of proteins or antibodies. The results of Fig. 2 indicate that, for our geometry, voltages of up to 700 V can be applied while still maintaining sufficiently low temperatures and achieving a reasonable total assay time of ~7 min. When higher temperatures are allowed, or perhaps even desired (*e.g*. for enhancing the specificity of hybridization assays), higher voltages can be applied, reducing the assay time to as little as 3 min at 1000 V.

### ITP focusing from large sample volumes

**Figure 3:**
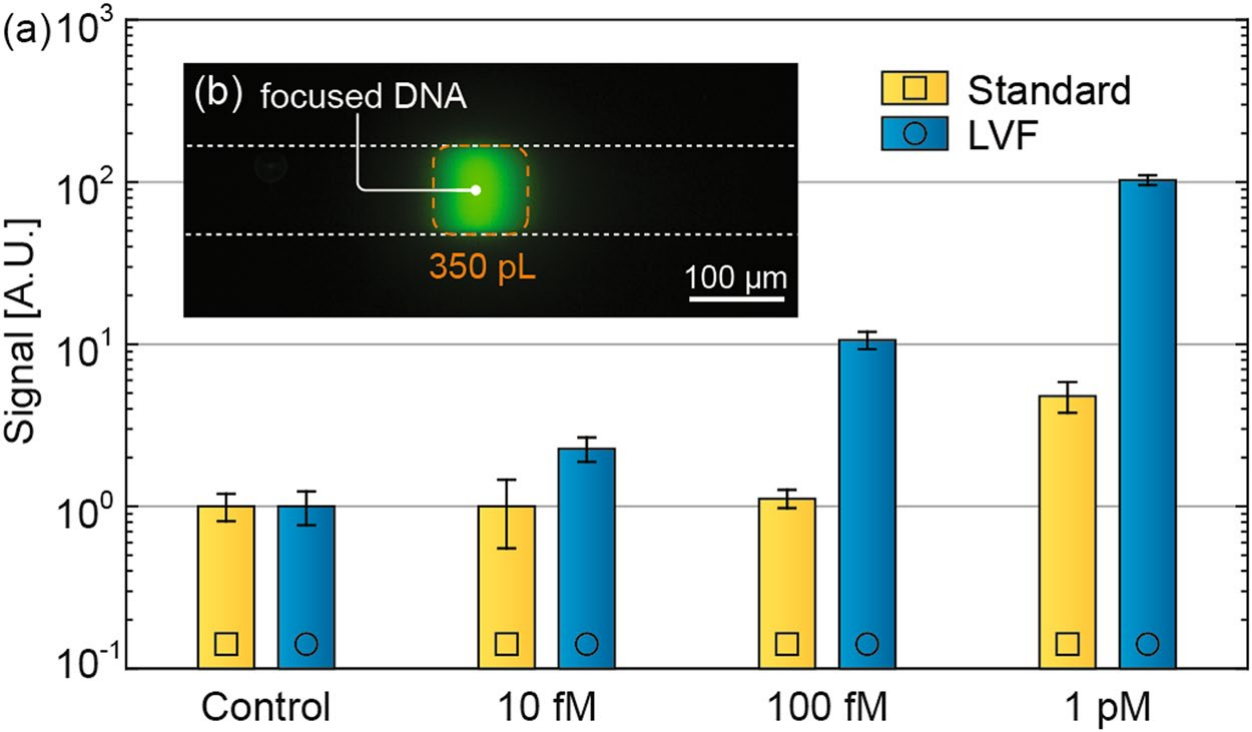
Experimental results of ITP focusing of Cy3-labeled DNA. **(a)** Comparison between focusing in a LVF chip and in a standard microchannel. The results show a minimum detectable concentration of 10 fM in the LVF chip, which is 2 orders of magnitude lower than in the standard microchannel. Due to differences in channel geometry between the LVF and the standard microchannel, we normalized the integrated signal values by the relevant control signals. Signal values should therefore only be compared within the same device. However, the LoD can be compared between devices, as it is a measure of the lowest detectable signal. Uncertainty bars represent the two-sided 95% confidence interval according to Student’s t-test based on 5 experiments per data point. **(b)** Fluorescence image of Cy3-labeled DNA focused into a plug with a volume of ~350 pL.

Figure 3 presents experimental results comparing the focusing of pre-labelled DNA in the LVF chip to focusing on a standard commercial chip (Caliper NS-95x, with a channel width of 35–75 μm, a depth of 12 μm and total length of 3.5 cm). We chose this chip as a benchmark, as it is commercially available, and widely used for on-chip CE experiments. The results presented show that the LVF chips enable a 310,000-fold increase in peak concentration (*n* = 4, std = 54,000): from an initial concentration of 10 fM to a peak concentration of 3.1 nM. This results in a 100-fold improvement in the limit of detection (LoD, defined as the lowest concentration giving a signal higher than three times the standard deviation of the negative control) as compared to standard NS-95x chips: from 1 pM in standard chips to 10 fM in LVF chips.

It is also instructive to compare the sample volume that has been processed in each of the chips. We found the processed volume by calculating the total mass contained in the ITP peak and dividing the resulting value by the initial concentration of the sample. This results in a processed volume of 49.7 μL (*n* = 4, std = 3.9 μL) in LVF chips, compared with 483 nL (*n* = 5, std = 110 nL) for the standard NS-95x chips.

While the peak concentration is a relevant figure of merit for direct detection assays, a more relevant figure of merit for binding assays is the mean concentration of the focused sample. The mean concentration, which we found by calculating the total mass contained in the ITP peak and dividing this by the volume of the focused plug, was increased in the LVF chip by a factor of 104,000 over the initial concentration in the reservoir (*n* = 4, std = 31,000).

### Application of LVF chips to homogeneous hybridization assays

**Figure 4:**
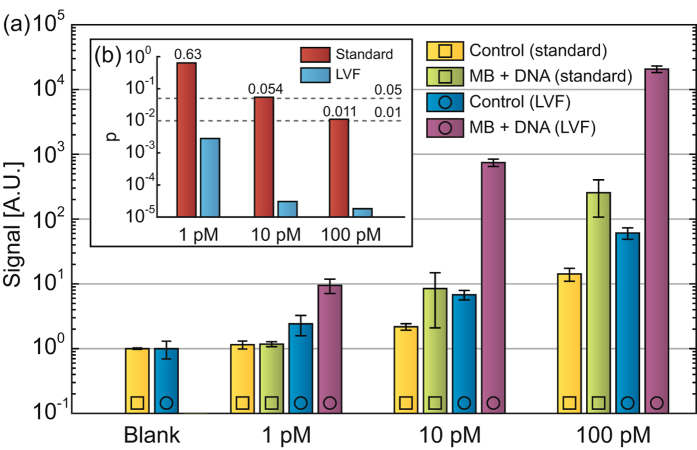
Experimental results comparing accelerated hybridization of molecular beacons (MBs) and DNA oligonucleotides in a large-volume focusing chip and in a standard glass chip. (**a**) Fluorescence intensity of MBs only (control) and of hybrids after 450 s. (**b**) The results of a paired t-test between the signal after hybridization and the signal of the control. The enhanced reaction kinetics in an LVF chip enable direct detection of 1 pM DNA with 1 pM MB (*p* = 2.8 × 10^−3^). Uncertainty bars represent the two-sided 95% confidence interval according to Student’s t-test based on 5 experiments per data point.

To study the potential benefits of the LVF chip for homogeneous hybridization assays (*i.e*., in the bulk), we performed DNA detection experiments at the ITP interface using molecular beacon (MB) probes^17^. Figure 4a shows the signal obtained from 450 s ITP hybridizations performed in either standard or LVF chips.

To characterize the ability of this detection method to distinguish between a hybridization signal and the control (containing only probes), Fig. 4b shows the p-value for a paired sample t-test between each signal and its control. The signal in LVF chips is significantly distinguishable from the control (*p* < 0.01) at 1 pM, whereas in standard chips a value of *p* < 0.01 is obtained only above 100 pM.

The relative importance of the hybridization reaction in the wide and narrow regions is determined by the transit time through either region, weighted by the channel width (which affects the concentration). Evidently, despite the short time (~10 s) spent in the narrow region by the focused target, the ~100-fold geometrical focusing makes this reaction time equivalent to approximately 1000 s of hybridization at lower concentrations in the wide region, ultimately enabling the low limit of detection.

### Application of LVF chips to focusing of bacteria

Armstrong *et al*.^18^ and Schneiderheinze *et al*.^19^ have demonstrated separation of bacteria using capillary electrophoresis, and focusing of bacteria has also been demonstrated, with Phung *et al*.^20^ detecting concentrations as low as 100 cfu/mL on a capillary electrophoresis apparatus. However, translation of the assays to an on-chip format has so far been limited by the volumes that could be processed; to enable on-chip detection at low concentrations, the processed sample volume must be sufficiently large to ensure that at least several of the bacteria present in the sample are focused by ITP.

**Figure 5:**
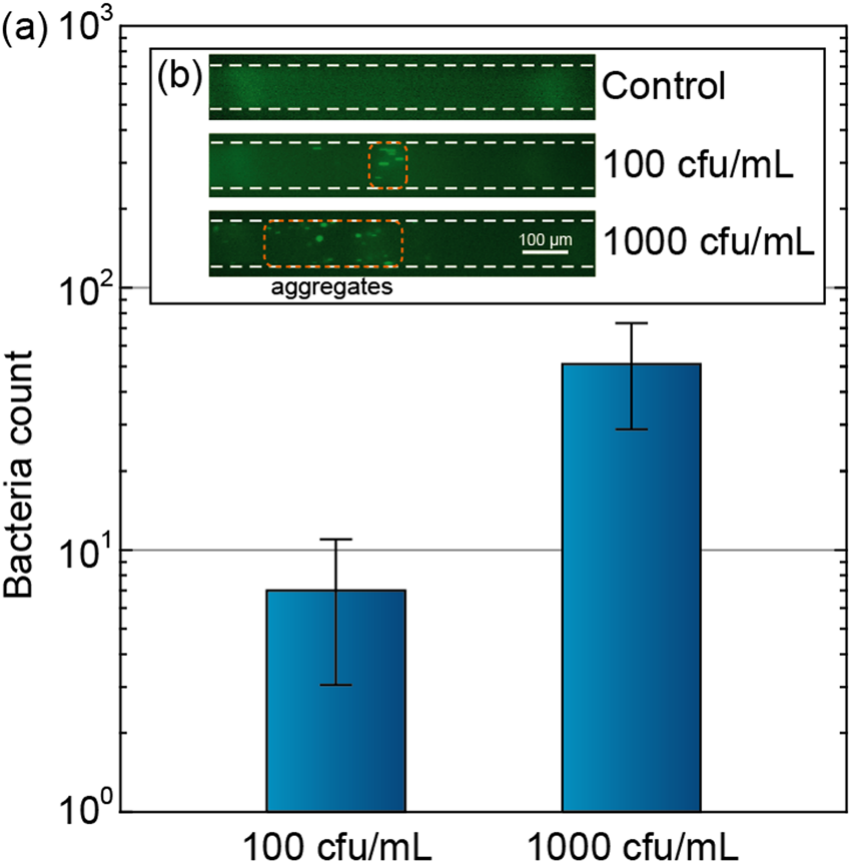
Experimental results demonstrating direct focusing of E. coli JM109 labelled with SYTO9 in our large-volume focusing chip. **(a)** From an initial concentration of 100 and 1000 cfu/mL, we respectively count ~7 and ~50 bacteria at the detection site (*n* = 5). **(b)** The bacteria are focused from an initial concentration of 100 cfu/mL into a compact plug with a final volume of 300 pL. Uncertainty bars represent the two-sided 95% confidence interval according to Student’s t-test based on 5 experiments per data point.

Figure 5 shows the number of bacteria counted at the ITP interface using our LVF chips, providing a limit of detection of 100 cfu/mL. From initial concentrations of 100 and 1000 cfu/mL, we recover respectively an average of 7 bacteria (*n* = 5, std = 3.1) and 50 bacteria (*n* = 5, std = 17.8), in approximately 7 min. This indicates a processed sample volume between 50 and 70 μL, which is in good agreement with the DNA focusing experiments.

At an initial concentration of 100 cfu/mL, the bacteria are focused into a final volume of ~300 pL. At higher initial concentrations (*e.g*. 1000 cfu/ml, see Fig. 5b) the bacteria appear to aggregate and the plug is more dispersed. Nevertheless, as the mobility of our TE and LE are far apart (*μ*_*T*_ = 11.3 × 10^−9^ m^2^/Vs, *μ*_*L*_ = 79 × 10^−9^ m^2^/Vs), focusing of both bacteria and aggregates is expected. We hypothesize that aggregates have different electrophoretic mobilities than single bacteria, causing them to focus at various ITP interfaces formed by additional species (*e.g*. carbonic acid^21^) which are naturally present in the sample.

## Conclusions

We presented a microfluidic chip for ITP focusing of analytes from 50 μL sample volumes. Our large-volume focusing chip consists of a wide region tapering down to a 100-fold narrower channel, and contains geometrical features designed to reduce dispersion arising from non-uniform entry of the ITP interface into the narrow region. This dispersion-limiting design allows the dimensions of the chip to be scaled up for processing larger volumes without loss of focusing.

The dimensions of the LVF chip are ultimately constrained by thermal considerations. In our LVF devices, the temperature remains below 40 °C at voltages of 700 V, enabling an assay time of 7 min. Further improvement in assay time may be achieved by etching the channels into thermally conductive substrates such as silicon (which must then be coated with a dielectric to prevent current leakage). Active cooling (*e.g*. by Peltier elements) may also be used, but results in a more complex system with a high energy consumption.

It has been shown that the, due to an increase in diffusivity with temperature, the length of the ITP interface is proportional to the ratio of the temperature to the electric field strength^10^. Given the quadratic dependence of temperature on the applied voltage, one would therefore expect the width of the ITP interface to increase with increasing electric field strength. We did not observe this in our experiments, possibly due to the fact that the aforementioned proportionality does not account for changes in mobility, dissociation constants and viscosity with temperature. As such, although diffusion is not a limiting factor when increasing the voltage in the LVF chips, it does play a role in broadening the ITP zone, and a higher maximum concentration may be achieved by actively or passively cooling the ITP system.

Using the LVF chip, we achieved 310,000-fold focusing, enabling direct detection of 10 fM DNA, a 100-fold improvement over standard glass microchannels. Importantly, in all experiments, we used standard buffers that are compatible with biological assays. In hybridization experiments, the strong focusing in the LVF chip accelerates the hybridization reaction, enabling a 1 pM limit of detection for unlabelled DNA (compared to 100 pM for the standard microchannel), despite the short (~10 s) reaction time at high concentrations. Multiplexed detection may be implemented by *e.g*. splitting the ITP interface^22^ to perform multiple assays in parallel, or by performing serial surface-based reactions on an array patterned on the wall of the channel^23^. Improved performance is expected with surface-based assays. Based on the results of Karsenty *et al*.^24^, Han *et al*.^23^ and Paratore *et al*.^25^ we expect a 10,000-fold lower limit of detection in LVF-based surface assays as compared to standard flow assays.

Finally, as a demonstration of the requirement of large-volume focusing for detecting discrete targets, we focused labelled bacteria and demonstrated detection of 100 cfu/mL. Although in our experiments the analyte was diluted in the TE, LVF devices are also compatible for focusing analytes present in the LE, which may be relevant to direct analysis (without *e.g*. filtration) of samples containing a high salt concentration. Detection of bacteria at concentrations of 100 cfu/mL is relevant for a range of infectious diseases (such as urinary tract infections^26^ (UTI)) and would not be possible using standard microfluidic chips. This demonstration of the capability to detect large, ‘discrete’ targets, as well as solute targets at low concentrations, is a step towards bridging the remaining gap in making on-chip ITP assays relevant for a range of clinical applications.

## Methods

### Device fabrication

We performed all ITP experiments either on our LVF chips or in ‘standard’ glass microchannels (NS-95x, Caliper, Waltham, MA), as indicated in each figure. For the fabrication of the LVF chips, we patterned a silicon wafer (Si-Mat, Kaufering, Germany) using conventional photolithography, before etching the exposed area using a Bosch process. We then stripped the resist and vapor-coated the wafer with trichloro(1H,1H,2H,2H-perfluorooctyl)silane (Sigma Aldrich, St. Louis, MO) in a desiccator and cast Sylgard 184 polydimethylsiloxane (PDMS, Dow Corning, Midland, MI) in a 10:1 ratio of base to crosslinker onto the wafer. After leaving the PDMS to cure for 2 hours at 80 °C, we peeled it off the wafer, and punched holes for the cathode (8 mm diameter) and anode (4 mm diameter) reservoirs. Finally, we treated the molded PDMS surface and a glass slide with oxygen plasma (30 s, 100 W) to irreversibly bond the two together.

### Assay protocols

#### Focusing experiments

We filled the entire channel with leading electrolyte (LE) consisting of 100 mM HCl (Merck, Darmstadt, Germany), 200 mM bistris and 1% w/v polyvinylpyrrolidone (PVP, Sigma Aldrich, St. Louis, MO; MW = 1.3 MDa). We then spiked Cy3-labeled 15-nt ssDNA (Sigma Aldrich, St. Louis, MO) directly into terminating electrolyte (TE) consisting of 10 mM tricine (Sigma Aldrich, St. Louis, MO) and 20 mM bistris (Sigma Aldrich, St. Louis, MO) and mixed it by brief vortexing. We rinsed the cathode reservoir with deionized (DI) water (Millipore, Billerica, MA), and filled it with the TE containing the DNA sample. Between experiments, we rinsed the channel with LE for 10 min.

#### Hybridization experiments

Hybridization experiments were performed similarly to the focusing experiments, but here we included 5 mM MgCl_2_ (Merck, Darmstadt, Germany) in the LE and sequentially spiked equal concentrations of molecular beacons (5′/5Cy5/CGAGCTCGTTTACRGCGTGGACTACCAGCTCG/3BHQ_2/3′, IDT, Coralville, IA) and complementary unlabeled target DNA (Sigma Aldrich, St. Louis, MO) into the TE.

#### Bacteria focusing experiments

We grew *Escherichia coli* culture (JM109 strain, a gift from Prof. Fishman, Technion) in Luria-Bertani (LB) broth at 37 °C with vigorous shaking, to an optical density of 0.3 at 600 nm (OD600), corresponding to approximately 1.8 × 10^8^ cfu/mL as measured by standard plating. We then concentrated the bacterial suspension by centrifugation at 14,000 × g for 2 min. Removing the supernatant, we resuspended the pellet in 0.85% NaCl and washed by an additional centrifugation step to remove significant traces of media. Next, we removed the supernatant and again resuspended the pellet in 0.85% NaCl, adding SYTO9 dye (L-7002, Molecular Probes, Eugene, OR) to a final concentration of 10 μM. The suspension was mixed and incubated at room temperature in the dark for 10 minutes. To discard remaining free fluorophores, we centrifuged the suspension, removed the supernatant and resuspended the pellet in TE to achieve the desired concentration. The composition of the LE was the same as in the focusing experiments, except for the concentration of bistris, which was 110 mM.

#### Temperature measurements

Temperature has a direct effect on ion solvation, the viscosity of the solution, and the dissociation constants of weakly ionized species, all of which affect the conductivity of the solution^27^. As in our device the resistance of the narrow channel dominates the overall device resistance, we found that conductance (or resistance) readings can serve as an excellent indirect measure of the temperature within the narrow channel. To construct a calibration curve, we fixed the chip to an indium tin oxide-coated glass heating plate (HI-57Dp, Cell Micro Controls, Norfolk, VA) controlled by a thermal controller (mTCII-HT, Cell Micro Controls, Norfolk, VA), and measured the conductance in an LVF channel filled with LE, by applying a low probing voltage (20 V) at fixed 10 °C intervals between 30 °C and 90 °C. We waited 5 min between measurements and confirmed that the temperature was stable to within 0.5 °C of the set point before reading the current.

### Fluorescence image processing

#### DNA focusing and hybridization experiments

We performed all DNA imaging with an upright microscope (AZ100, Nikon, Tokyo, Japan) equipped with a 5× objective (NA 0.5, AZ Plan Fluor, Nikon, Tokyo, Japan) set to 3× optical zoom, an LED light source (Sola, Lumencor, Beaverton, OR) and a CMOS camera (Zyla, Andor, Belfast, UK). We used a Cy3 filter (TRITC-B-NTE, Semrock, Rochester, NY) for observing labelled DNA (focusing experiments), a Cy5 filter (49006, Chroma, Bellows Falls, VT) for detecting molecular beacons (hybridization experiments).

In the LVF chips, we chose a detection point 1.4 cm downstream from the end of the converging region. For the DNA focusing experiments, we calculated the final (focused) concentration from a calibration curve constructed by filling the channel with known concentrations of DNA solution. The total amount of focused sample could then be found by spatially integrating over the resulting concentration map and multiplying by the depth of the channel. In the integration, we used a threshold of 10% of the peak value. We confirmed that no cross-contamination occurred between runs by comparing the fluorescence signature after ITP focusing in a new device in the absence of DNA to that in a device previously used to focus a high concentration of DNA. For the hybridization experiments we calculated the signal by integrating over a fixed 100 × 200 μm^2^ region in the channel.

#### Bacteria focusing experiments

We imaged the bacteria on an inverted microscope (Eclipse Ti-U, Nikon, Tokyo, Japan) equipped with a 10× objective (NA 0.45, WD = 4 mm, AZ Plan Apo, Nikon), an Intensilight C-HGFI light source (Nikon, Tokyo, Japan) and a Neo sCMOS camera (Andor, Belfast, UK). We used a FITC filter (49011, Chroma, Bellows Falls, VT) for SYTO9 (bacteria focusing). In the LVF chips, we chose a detection point 1.4 cm downstream from the end of the converging region. We manually counted the bacteria over 5 frames to account for any bacteria moving vertically into and out of the focal plane.

### Data availability

The datasets generated during and/or analysed during the current study are available from the corresponding author on reasonable request.

## Electronic supplementary material

SI Video S1

Supplementary Information
