# Supplementary material for: Focusing analytes from 50 μL into 500 pL: On-chip focusing from large sample volumes using isotachophoresis
Source: Sci Rep. 2017 Sep 5;7:10467. doi: 10.1038/s41598-017-10579-5 (PMC5585209; doi:10.1038/s41598-017-10579-5)
Supplement: Supplementary file 2 — Supplementary Information [file 41598_2017_10579_MOESM2_ESM.pdf]

Supplementary Information

# Focusing analytes from 50 µL into 500 pL: On-chip focusing from large sample volumes using isotachophoresis

X.F. van Kooten[a,b], M. Truman-Rosentsvit[a], G.V. Kaigala*[b] and M. Bercovici*[a]
[a]Technion – Israel Institute of Technology, Haifa, Israel
[b]IBM Research – Zurich, Rüschlikon, Switzerland

## S1. Pillar arrays

Figure S1 presents a comparison of the shape of the ITP interface in the wide channel, with and without the pillar array. We fabricated the pillar-free channel in a 300 µm PDMS layer and bonded the top side to a glass slide which acted as an external structural support to prevent the collapse of the channel's 'ceiling'. Fig. S1a shows the lateral non-uniformities of the ITP interface that occur in the absence of pillars. We hypothesize that these disturbances (which also oscillate in time) are related to electrokinetic instability of the interface. The addition of pillar structures results in a highly uniform and stable propagation of the interface (Fig. S1b).

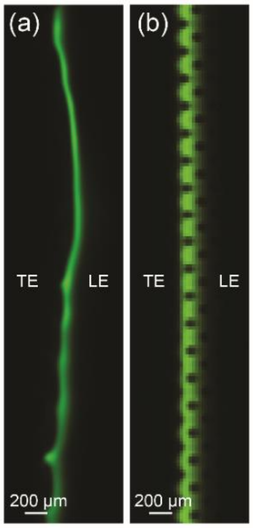


***Figure S1.*** *Experimental comparison of the ITP interface in the wide region of the LVF chip, in the absence and presence of pillars.* ***(a)*** *In the absence of pillars, we observed significant oscillations of the ITP interface, together with non-uniformity in the lateral direction.* ***(b)*** *Adding a pillar array to the wide region contributes to viscous resistance, resulting in a straight interface throughout the channel.*

To determine the required pillar distribution, we fabricated several pillar configurations and evaluated their performance by focusing Dylight 650 NHS (Thermo Fischer, Waltham, MA) and observing the uniformity and symmetry of the focused dye across the width of the channel. Of the configurations shown in Fig. S2, we obtained the best performance using a staggered array positioned at an angle of 45° with respect to the electric field. Of the configurations with the same angle, smaller pillar diameters ($D = 50\ \mu m$) and pitch ($P = 100\ \mu m$) led to better uniformity than the larger pillars with larger pitch ($D = 100\ \mu m, P = 200\ \mu m$), and were therefore selected for this work.

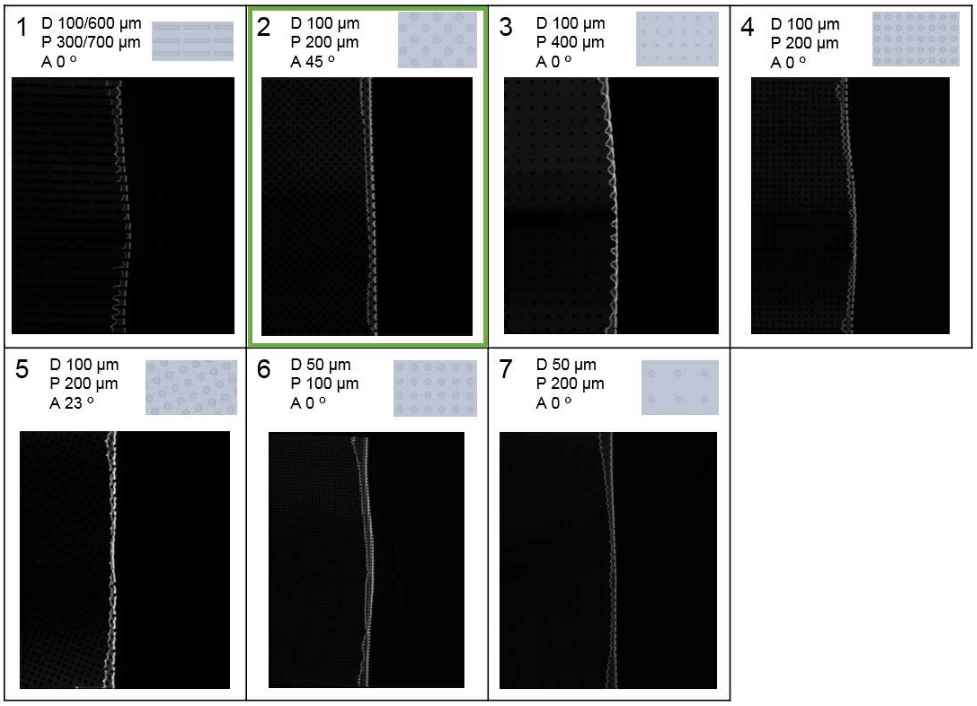


***Figure S2.*** *Experimental results showing ITP focusing in the wide region for pillar configurations with varying diameter (D), center-to-center pitch (P) and angle w.r.t. the electric field (A). We used a design with* $D = 50\ \mu m, P = 100\ \mu m$ and $A = 45°$ *for our experiments, as this was found to give the most uniform ITP interface. The broad (dual) peaks that appear when focusing Dylight 650 NHS highlights any non-uniformities along the interface, such as in geometry 1.*

## S2. ITP interface in converging channels

The tapering angle of the converging region between the wide and narrow section of the channel must be chosen to minimize dispersion of the ITP interface while maximizing the internal volume of the chip. Fig. S3 shows the geometries tested to this end, with a tapering angle $\theta$ between 10° and 60°. While lower tapering angles are preferred as they cause less sample dispersion, they lead to a smaller internal volumes, and therefore reduce the processed sample volume. Conversely, higher tapering angles enable a larger

internal volume, but lead to more dispersion. We found that geometries with an intermediate tapering angle $\theta = 30°$ provided a good trade-off between dispersion and internal volumes.

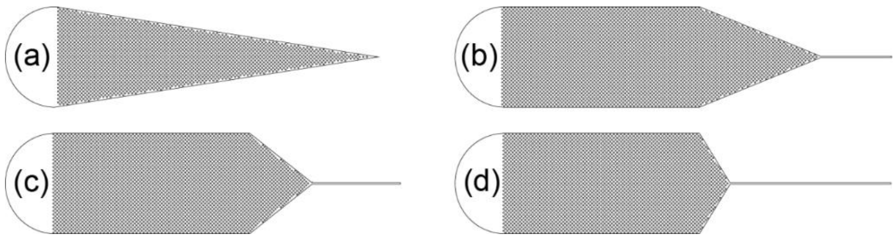


***Figure S3.*** *Top-view schematics of devices with different tapering angles* $\theta$ *of the converging region.* ***(a)*** $\theta = 10°$ ***(b)*** $\theta = 30°$, ***(c)*** $\theta = 40°$, ***(d)*** $\theta = 60°$. *Lower values of* $\theta$ *are less preferred due to the limited internal volume, while higher values of* $\theta$ *enable large internal volumes but may adversely affect focusing in the converging region. We observed the best performance with* $\theta = 30°$.

While Fig. S1 shows the need for pillars in the wide region, we found that a similar requirement holds in the converging region. Fig. S4a illustrates how, in the absence of pillars (which we tested using a very sparse array of narrow pillars), the ITP interface enters the narrow channel asymmetrically, resulting in dispersion in the narrow channel. Adding a dense array of larger pillars (Fig. S4b) greatly improves the robustness of focusing and consistently results in symmetric focusing. However, dispersion is not completely eliminated, as some of the sample closer to the sidewalls still enters the narrow channel before the rear of the interface (Fig. S4c).

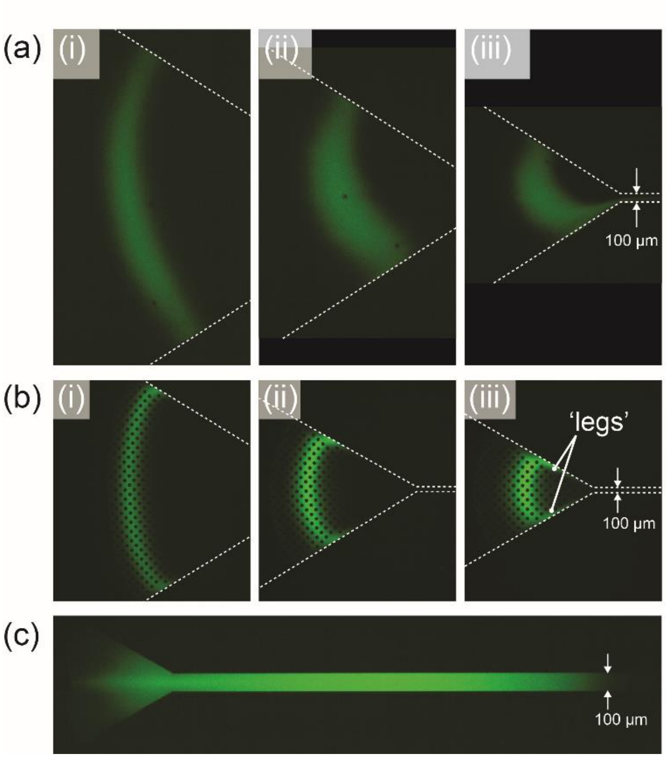


***Figure S4.*** *Fluorescence images of the ITP interface at the boundary between the converging and narrow region, in the presence and absence of pillars.* ***(a)*** *With very sparse pillars (which act only as structural support and can be approximated as the absence of pillars) the ITP interface typically progresses asymmetrically, so that one side reaches the narrow channel before the other. Different parts of the ITP interface reach the narrow region at different times, leading to elongation of the interface and dispersion over length scales from which re-focusing within the length of the narrow channel in not feasible.* ***(b)*** *This asymmetry can be corrected by adding an array of pillars to the channel.* ***(c)*** *'Legging' of the sample as it enters the narrow channel still results in significant dispersion, but a focused interface can be recovered, e.g. by adding a chamber geometry to the narrow region.*

The dispersion due to 'legs' can be overcome by adding geometrical features near the entrance to the narrow channel. Fig. S5 shows several designs with such features, which include moderate tapering angles and step-wise tapering. A lower tapering angle at the end of the converging region (Fig. S5c) did not reduce dispersion. Step-wise tapering (Fig. S5d) largely prevented dispersion, but was found to be less robust than the chamber geometry (Fig. S5e-h), where the channel widens to slow down the fast moving front, while allowing the rear part of the interface to close the gap. Varying the length of the chamber changes the time allowed for the interface to recover. In the short chamber (Fig. S5g) the interface does not become uniform ('straight') before reaching the end of the chamber. The best performance was achieved with a 3 mm long

chamber incorporating a dense pillar array (Fig. S5h), followed by the 1 mm long chamber with a sparse pillar array (Fig. S5e).

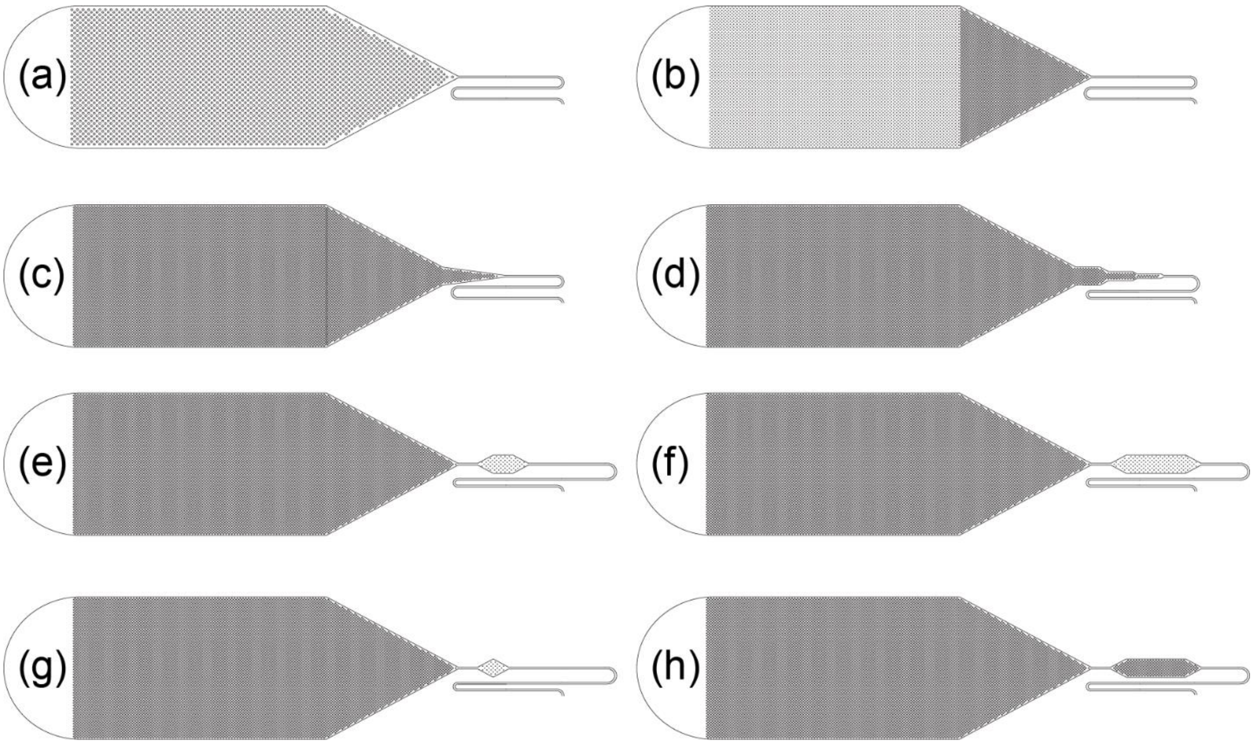


***Figure S5.*** *Top-view schematics of the large-volume focusing geometries tested, which include geometrical features before the entrance to the narrow channel, aimed at recovering a focused interface from its dispersed state at the end of the converging region.* ***(a)*** *Control with a sparse pillar array,* ***(b)*** *Sparse pillar array in the wide region and dense pillar array in the converging region,* ***(c)*** *Gradual tapering,* ***(d)*** *Step-wise tapering,* ***(e)*** *1 mm long chamber with sparse pillars,* ***(f)*** *3 mm long chamber with sparse pillars,* ***(g)*** *diamond chamber with sparse pillars,* ***(h)*** *3 mm chamber with dense pillars. We achieved the best performance with configurations (h) and (e).*

***Table S1.*** *Peak fluorescence intensity and width of the ITP interface for each of the devices in Fig. S5, measured 1.4 cm downstream of the first entry into the narrow channel. We define the width as the distance between the locations at which the signal drops to 10% of its peak value. Design (h) consistently showed the best performance, both in terms of peak fluorescence intensity and interface width.*

| | Experiment 1 | | Experiment 2 | |
|---|---|---|---|---|
| Device in Fig. S5 | peak [A.U.] | width [px] | peak [A.U.] | width [px] |
| a | 1200 | 1400 | 500 | 3200 |
| b | 900 | 1700 | – | – |

| | | | | |
|---|---|---|---|---|
| c | 2100 | 650 | 1200 | 1050 |
| d | 2500 | 675 | 2200 | 790 |
| e | 6500 | 200 | 5500 | 190 |
| f | 2900 | 300 | 3000 | 390 |
| g | 1500 | 630 | 800 | 2000 |
| **h** | **8400** | **140** | **6500** | **180** |

## S3. Electrode placement

As is described by Persat, Suss and Santiago[1], the placement of electrodes in the reservoir of an electrophoresis chip may affect the focusing result. Two general recommendations for electrode placement are given: "(i). the wire is kept well away from the channel entrance to mitigate the effects of pH changes and bubbles generated by the electrode; and (ii). placing the tip of the wire at the bottom of the reservoir is easier to reproduce (vs. suspending the wire part way down the reservoir)."[1]

We heeded these recommendations by using x,y,z-micropositioners (SE40, Perfict Lab, Shenzhen, China) with a custom electrode holder to position the platinum electrodes (diameter 0.5 mm) in a reproducible way. The electrodes were always placed vertically along the wall of the reservoir diametrically opposite the channel entrance, and the z-stage was adjusted so that the electrode came in contact with the floor of the reservoir.

## S4. Approximation of constant current during electromigration through the wide channel section

For an LVF channel of depth $H_c$, comprising a wide-channel section of effective width $w_{w,eff}$ and length $l_{w,eff}$ (both accounting for the occlusion of the channel by pillars) filled with an electrolyte with conductivity $\sigma_w$, connected in series to a narrow-channel section of width $w_n$ and length $l_n$ containing an electrolyte with conductivity $\sigma_n$, the ratio of the electrical resistances of the wide and narrow channel is $R_w/R_n = \sigma_n w_n l_{w,eff}/\sigma_w w_{w,eff} l_n$.

Initially, the whole channel is filled with LE, $\sigma_n = \sigma_w = \sigma_{LE}$ and $R_w/R_n = w_n l_{w,eff}/w_{w,eff} l_n$. With the dimensions of the tested LVF chips, $l_{w,eff} = 8.2$ mm, $l_n = 2$ cm, $w_{w,eff} = 5.25$ mm and $w_n = 100\ \mu m$, we find $R_w/R_n = 0.0078$, and the resistance of the wide region can be safely neglected.

As the ITP interface progresses into the channel, the contribution of the wide region to the total channel resistance increases. At this point, $\sigma_n = \sigma_{LE} = 1.01$ S/m (for LE consisting of 200 mM bistris, 100 mM HCl) and $\sigma_w = \sigma_{TE,adjusted} = 0.07$ S/m (for TE consisting of 20 mM bistris, 10 mM tricine), so that $R_w/R_n = 0.11$. This is the maximum contribution of the wide region to the overall resistance of the

channel, as further progression of the ITP interface would lead to low-conductivity TE entering the narrow region, once again increasing the voltage drop over the narrow channel. Therefore, it can be safely assumed that the voltage drop over the wide region never exceeds 15% of the applied voltage. Using the approximation that the resistance of the narrow channel dominates the overall resistance, the current during electromigration of the interface through the wide region can be expressed as $I = V w_n H_c \sigma_{LE} / l_n$.

## S5. Thermal equilibrium in the LVF chip

Joule heat generated in the narrow channel is transferred downwards through the glass substrate to the microscope stage, and upwards to the surface of the PDMS. Following a similar analysis as presented by Zehavi *et al.*[2], we consider convection at the upper surface of the PDMS and use an isothermal condition at the lower surface of the glass slide, which is in contact with the glass microscope stage and can therefore be considered to be at room temperature. The combined thermal resistance of the PDMS on top of the channel and of free convection in air is $R_{PDMS} = d_{PDMS}/k_{PDMS} + 1/h$ = 0.12 $m^2KW^{-1}$ (for $h$ = 10 $Wm^{-2}K^{-1}$, $d_{PDMS}$ = 3 mm, and $k_{PDMS}$ = 0.15 $Wm^{-1}K^{-1}$)[3], and through the glass bottom, in contact with to the microscope stage, $R_{glass} = d_{glass}/k_{glass}$ = $1\times10^{-3}$ $m^2KW^{-1}$. Since $R_{PDMS}$ and $R_{glass}$ are parallel thermal resistances, heat conduction through the glass substrate is dominant. Further, since $d_{glass}^2/\alpha$ = 2 s is much shorter than the typical experiment time, we may assume a steady state and the heat equation can be simplified to:

$$Q = \sigma_{LE} E^2 = \frac{\Delta T}{H_c} \frac{1}{R_{glass}}$$

where $\Delta T$ is the difference between the temperature in the channel and the ambient. Rewriting this, and using the approximation that the narrow channel accounts for most of the voltage drop, we obtain:

$$\Delta T = \frac{\sigma_{LE} H_c d_{glass}}{k_{glass}\, l_n^2} V^2.$$

## References


(1) Persat, A.; Suss, M. E.; Santiago, J. G. *Lab Chip* **2009**, *9* (17), 2454–2469.
(2) Zehavi, M.; Boymelgreen, A.; Yossifon, G. *Phys. Rev. Appl.* **2016**, *5* (4), 044013.
(3) Incropera, F. P.; DeWitt, D. P.; Bergman, T. L.; Lavine, A. S. *Fundamentals of Heat and Mass Transfer*, 7th ed.; John Wiley & Sons.
